# Supplementary figures and images for: Disease Mutation Study Identifies Critical Residues for Phosphatidylserine Flippase ATP11A
Source: Biomed Res Int. 2020 Jun 2;2020:7342817. doi: 10.1155/2020/7342817 (PMC7288202; doi:10.1155/2020/7342817)

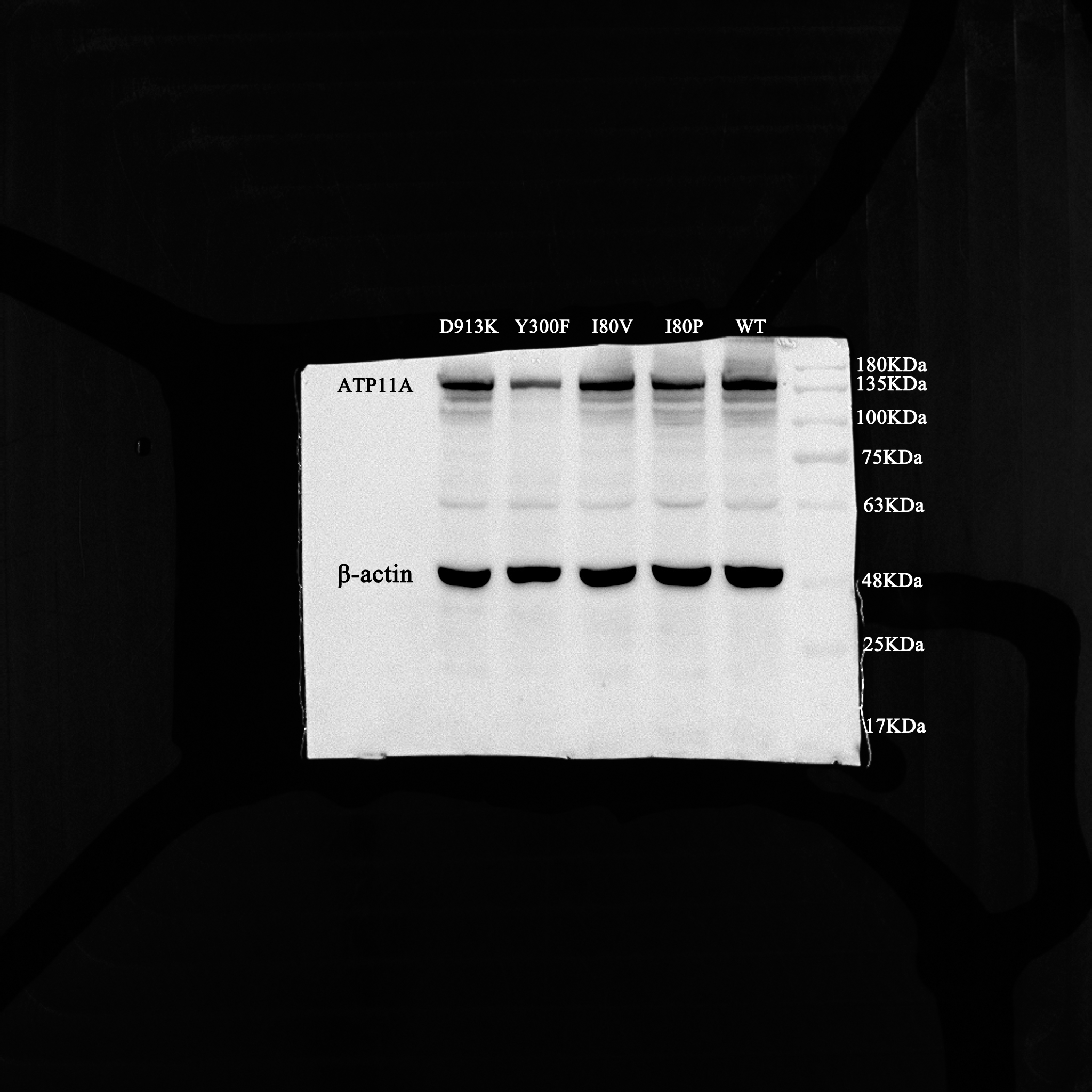

Supplement: Supplementary Materials — Figure S1: the uncropped gel images for Figure 2. [file 7342817.f1.tif]
